# Supplementary material for: Association between oral microbiome and seven types of cancers in East Asian population: a two-sample Mendelian randomization analysis
Source: Front Mol Biosci. 2023 Nov 21;10:1327893. doi: 10.3389/fmolb.2023.1327893 (PMC10702768; doi:10.3389/fmolb.2023.1327893)
Supplement: Supplementary file 1 [file DataSheet1.ZIP › Supplementary Materials/MR plots of saliva/saliva═╝/ovarian cancer/pheno.858_to_ovarian cancer_forest.pdf]

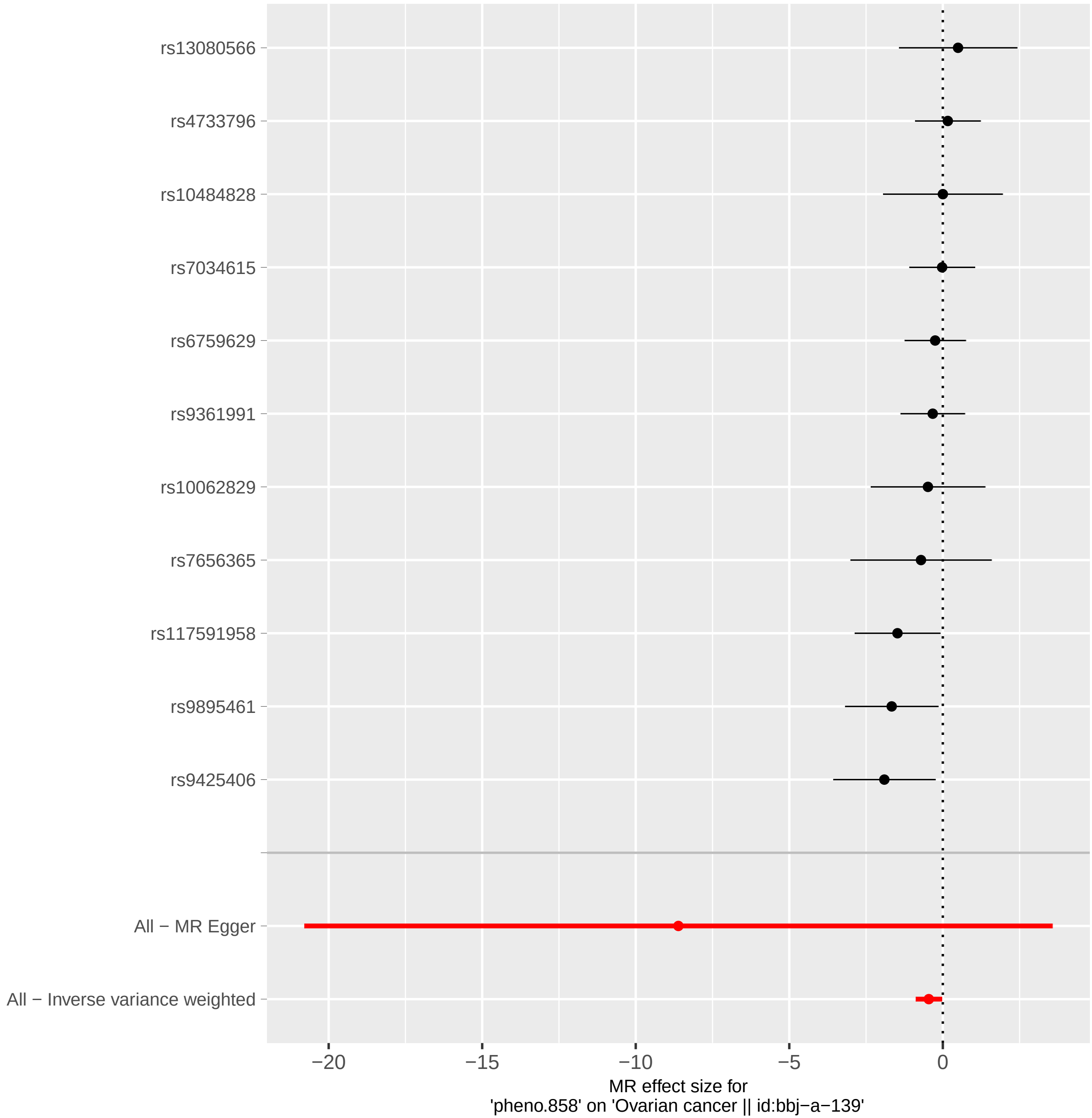
rs13080566
rs4733796
rs10484828
rs7034615
rs6759629
rs9361991
rs10062829
rs7656365
rs117591958
rs9895461
rs9425406
All − MR Egger
All − Inverse variance weighted
−20
−15
−10
−5
0
MR effect size for
'pheno.858' on 'Ovarian cancer || id:bbj−a−139'
